# Response score-based protein structure analysis for cancer prediction aided by the Internet of Things

**DOI:** 10.1038/s41598-024-52634-y

**Published:** 2024-01-28

**Authors:** Omar Alruwaili, Amr Yousef, Touqeer A. Jumani, Ammar Armghan

**Affiliations:** 1 Department of Computer Engineering and Networks, College of Computer and Information Science, Jouf University, 72388 Sakaka, Saudi Arabia; 2 Electrical Engineering Department, University of Business and Technology, 23435 Ar Rawdah, Jeddah, Saudi Arabia; 3 Engineering Mathematics Department, Alexandria University, Lotfy El-Sied St. Off Gamal Abd El-Naser, Alexandria, 11432 Egypt; 4 Department of Electrical Engineering, Mehran University of Engineering and Technology, SZAB Campus, Khairpur Mirs, 66020 Pakistan; 5 Department of Electrical Engineering. College of Engineering, Jouf University, 72388 Sakaka, Saudi Arabia

**Keywords:** Cancer, Engineering, Physics

## Abstract

Medical diagnosis through prediction and analysis is par excellence in integrating modern technologies such as the Internet of Things (IoT). With the aid of such technologies, clinical assessments are eased with protracted computing. Specifically, cancer research through structure prediction and analysis is improved through human and machine interventions sustaining precision improvements. This article, therefore, introduces a Protein Structure Prediction Technique based on Three-Dimensional Sequence. This sequence is modeled using amino acids and their folds observed during the pre-initial cancer stages. The observed sequences and the inflammatory response score of the structure are used to predict the impact of cancer. In this process, ensemble learning is used to identify sequence and folding responses to improve inflammations. This score is correlated with the clinical data for structures and their folds independently for determining the structure changes. Such changes through different sequences are handled using repeated ensemble learning for matching and unmatching response scores. The introduced idea integrated with deep ensemble learning and IoT combination, notably employing stacking method for enhanced cancer prediction precision and interdisciplinary collaboration. The proposed technique improves prediction precision, data correlation, and change detection by 11.83%, 8.48%, and 13.23%, respectively. This technique reduces correlation time and complexity by 10.43% and 12.33%, respectively.

## Introduction

Cancer prediction is an important task to perform in every healthcare center. The cancer prediction method predicts the exact types of cancer-based on features and factors. Protein structure analysis is a process that analyzes the actual amino acid sequence or structure of a person^1^. The amino acid structures are identified using an electron microscope and spectroscopy scans. Protein structure analysis-based cancer predictions are commonly used to improve the accuracy of the prediction process^2^. An effective cancer driver gene prediction method based on protein structure is used to predict the cancer types of the patients. The goal is to predict cancer genes using biological information^3^. The biological information contains the exact structure of amino acids, which also provides features for the prediction process. The cancer prediction method increases accuracy, which improves the feasibility of further medical diagnosis processes^4^. A deep multimodal prediction method is also used for cancer-type prediction. The multimodal prediction identifies the protein–protein interactions among amino acids, which produce optimal data for prediction. The deep multimodal method reduces the complexity of the cancer prediction process^5,6^.

Internet of Things (IoT) technology is commonly used in many fields to improve the functional capability of systems. IoT-based computation is used for the cancer prediction process^7^. IoT-enabled cancer prediction framework predicts patients’ cancer levels and condition. The IoT-enabled framework uses a computer-aided diagnosis system (CAD), which provides feasible information for cancer prediction^8^. The CAD calculates the features and patterns of cancer via IoT. The user’s health condition and texture of cancer level are evaluated based on the biological characteristics of the patients. The IoT-enabled framework increases the efficacy and reliability range of healthcare systems^9^. IoT creates a significant impact on the cancer prediction computation process. IoT is mainly used to solve the difficulty and latency in cancer prediction^10^. IoT-based data analytic technique is used to track or detect cancer-affected patients. IoT-based technique gathers the relevant information from protein structure, which predicts cancer-related symptoms for prediction. Ensemble technique, that draws on ML, to identify the most important elements influencing the occurrence of gastric cancer. Several machine learning approaches, such as ridge regression, elastic net, logistic regression, and random forest. The IoT-based technique provides effective computation services that enhance the performance range of the diagnosis process^11,12^.

Inflammatory response score computation for protein structure change is crucial in cancer prediction. Differentially expressed protein (DEP) and other protein changes are identified using biological information for the patients^13^. The actual aim is to predict the response scores of the patients. Total responders (TR) and poor responders (PR) are predicted based on DEP features^14^. A functional enrichment analysis technique analyses protein changes’ characteristics and operating features. The analysis technique understands the features of amino acids, which reduce the energy consumption level in the computation process^15,16^. An ensemble residual convolutional neural network (RCNN) algorithm-based cancer prediction method analyzes the protein structural changes. Protein–protein interaction (PPI) variations, which provide feasible data for cancer prediction, are collected. The RCNN recognizes the exact changes that occur in protein structure. The RCNN-based method increases the accuracy of the cancer prediction process^17,18^.

The contributions are listed below:

- Designing a sequence-based cancer prediction using the folding structure of proteins and inflammatory response scores
- Performing a data correlation using inflammatory score with the clinical correlation using ensemble learning for structural change prediction
- Performing a data-based validation and comparative analysis using external datasets, methods, and metrics

## Related works

Liu et al.^19^ introduced a network-based cancer driver gene prediction classification method. The presented method identifies the critical data using biological information of the patients. The biological information produces important detection features that reduce the prediction process’s latency. The introduced method also used the protein–protein interactome, which provides appropriate data for the cancer classification process. The actual types and textures of the cancers are identified for the diagnosis process.

Abdollahi et al.^20^ proposed a cancer-associated protein–protein interaction (PPI) based cancer prediction model. A deep learning algorithm is used in the model to understand the biological features of cancer. The PPI is mainly used here to identify the necessary features, such as behavioral features of cancer symptom patients. The proposed model increases the accuracy of cancer prediction, enhancing the healthcare system’s performance range.

Albu et al.^21^ designed a deep multimodal stacked generalization approach for PPI prediction. A graph attention network is implemented to sequence the information for PPI prediction. The attention network gathers information from trained proteins, reducing the energy consumption level in the PPI prediction process. It improves the effective range of cancer prediction and diagnosis processes. Experimental results show that the designed approach enhances the generalization of PPI.

Berrino et al.^22^ proposed a unique pattern recognition method for mismatch repair (MMR) protein expression in colorectal cancer (CRC). DNA is used in the method, which is extracted using MMR. Immunohistochemistry (IHC) testing is implemented to identify the relevant CRC features for the detection process. The proposed method identifies the exact MMR protein expression for IHC testing. The proposed method increases the sustainability and feasibility level of CRC prediction systems.

Chuang et al.^23^ introduced a convolutional neural (CNN) based approach for cancer-type prediction. PPI network is also used in the approach, which generates two-dimensional (2D) images for cancer prediction. The 2D images contain necessary cancer features and patterns that reduce the latency in further type prediction processes. Compared with other approaches, the introduced approach maximizes the accuracy range in the cancer-type prediction process.

Elwahsh et al.^24^ developed a deep neural learning (DNL) cancer prediction approach. The main aim of the approach is to predict the actual types of cancers. The approach uses a feature selection technique that selects what is necessary for classification and prediction processes. It also classifies the types of cancer based on the extracted features that enhance the energy-efficiency level of the systems. The developed DNL-based approach increases the accuracy of cancer prediction, improving the healthcare systems’ performance range.

Sattar et al.^25^ proposed using a multi-gene genetic programming algorithm to predict lung cancer. The algorithm is mainly used here to gather biological information’s protein amino acid ratio. It is used as an evolutionary learning technique that identifies the actual types of lung cancer. The programming algorithm reduces both time and energy consumption levels in the computation process. The proposed prediction method improves the performance and reliability range of the lung cancer diagnosis process.

Zhang et al.^26^ designed a new cancer diagnosis method using micro-coding RNA (miRNA) and long non-coding RNA (lncRNA). A feature selection algorithm, which detects the relationship between miRNA and lncRNA, is used in the method. The designed method uses a heterogeneous classification model to classify the types of cancer. The optimization problems that occur during the computation process are solved. The developed method increases the effectiveness of the cancer classification process.

Cheng et al.^27^ proposed a deep neural network (DNN) based lung cancer prediction method. The blood pressure, gene expressions, and deterioration of the tumor level are predicted from PPI-generated images. The analyzed information provides the necessary information for the lung cancer prediction process. The essential genes of the tumor are also identified using an analysis process. Experimental results show that the proposed method improves the accuracy of lung cancer prediction, improving the diagnosis’s feasibility.

Zuo et al.^28^ introduced a new deep-learning model for drug response prediction. The main aim of the model is to predict the compound activities of the patients. The genomics of drug sensitivity in cancer (GDSC) is implemented in the model, which selects relevant features for the prediction process. The GDSC identifies the exact cheminformatics features for further processes. The introduced model improves the performance range of the healthcare systems.

Lei et al.^29^ proposed a stacking model to predict compound-protein binding affinity (CPA). CPA prediction is a complicated task in drug response prediction, requiring optimal information for the prediction process. The proposed model detects the critical protein level from the pocket, which reduces the latency in CPA prediction. The atomic level, subdomain level, and residue level of the binding region are also identified for further processes. The proposed model improves the effective range of the drug response prediction process.

Ali et al.^30^ introduced a machine learning (ML) cancer driver gene prediction method. An artificial neural network (ANN) algorithm, which identifies the essential characteristics and features for gene prediction, is used in the method. ANN validates the features collected from numeric data and reduces optimization problems in the computation process. Compared with other methods, the introduced method increases the accuracy level in the gene prediction process (Table 1).

**Table 1:** Summary of related works.

Ref. No.	Key techniques	Dataset used	Advantages	Disadvantages
^19^	network-based driver gene prediction	Patient data	Critical data identification	Limited discussion on methodolgy
^20^	cancer-associated protein–protein interaction (PPI)	PPI data	Improved accuracy	Limited explanation on deep learning
^21^	deep multimodal stacked generalization approach for PPI	Trained protein data	Reduced energy consumption	Limited graph attention
^22^	MMR-CRC	DNA-immunohistochemistry (IHC) testing	Sustainability in CRC prediction	Lack of detailed MMR
^23^	CNN based approach	PPI network	Improved accuracy	Lack of protein sample structure
^24^	DNL cancer prediction	Clinical data samples	Improved energy efficiency	Lack of feature selection technique
^25^	multi-gene genetic programming algorithm	Biological information’s protein amino acid ratio	Reduced time and energy consumption	Limited information on genetic progression
^26^	miRNA and lncRNA	Three EPs of miRNA, lncRNA and PCG in database of the cancer genome atlas (TCGA)	Solves optimization problems	Complexity due to bigger dataset
^27^	DNN based lung cancer prediction	Blood pressure, gene expressions, and deterioration of the tumor level	Enhanced prediction accuracy	Increased complexity due to repeated sampling
^28^	deep-learning model	Genomics of drug sensitivity in cancer (GDSC)	Improved overall performance	complex SWnet need to be well-trained for larger dataset
^29^	stacking model for CPA prediction	Case study for epidermal growth factor receptor erbB1 (EGFR)	Reduced latency	similarity of the protein subdomain is unconsidered
^30^	ML-ANN	genetic data with biological functions	Increased accuracy in gene prediction	needs more time to extract robust features
^31^	Anti inflammatory peptide with self normalized temporal convolutional network (AIPs-SnTCN)	Wet laboratory treatments for inflammation	Improved accuracy	Complex designing model
^32^	Antifreeze proteins consensus multi-blocks position (AFP-CMBPred)	Pfam database	Higher prediction accuracies	Lack of species information regarding drug development model

The protein structure is used to detect the level of cancer and then the number of cancer cells in the human body. Using 3D to enhance the precision improvements helps in the detection process. The peculiarity of the protein structure leads to defective cellular procedures, contributing to the enhancements and succession of the cancer cells. The 3D arrangements of the protein structure help determine the critical structural changes associated with cancer, namely, mutations, dysregulation, and altered interactions in the protein structures. These variations in the protein structures help in cancer detection and diagnosis. This protein structure helps estimate the developed mechanisms to cure cancer cells and helps create therapies that extract these protein structural abnormalities.

### Data briefing

The data from the^33^ source is extracted for analyzing the proposed technique. The information on 890 lung cancer patients is utilized based on inflammation scores (1, 2, and 3). This information was obtained between 2018 and 2019; the scores are computed using the conditions in Table 2.

**Table 2:** Inflammatory score.

Prognosis	Condition (g/L)	Score	Results	
C-reactive protein (CRP)	$\le 8$	1	✓	✓
Albumin	$\ge 35$		✓	✓
C-reactive protein (CRP)	$\le 8$	2	✗	✓
Albumin	$\ge 35$		✓	✗
C-reactive protein (CRP)	$\le 8$	3	✗	✗
Albumin	$\ge 35$		✗	✗

The above tabulation presents three scores based on g/L condition using clinical values of the Glasgow prognostic score. The above values are inherited for analysis for 18 observations in a 5-day interval sequence in the proposed technique validation. Though different methods are used for score detection, the range from 1/3 to 3/3 (i.e.) min score/max score to max/ max score is computed along the sequence for calculating the inflation response progress (Refer to Table 1).

## Protein structure prediction technique based on three-dimensional sequence

**Figure 1:**
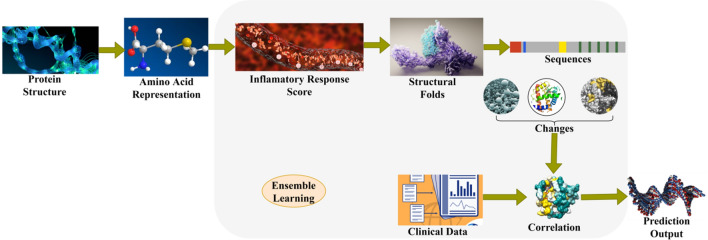
PSPT complete functional representation.

Assimilating modern technologies such as the Internet of Things (IoT) has substantially improved medical recognition and research, especially in cancer. One dimension that has a vital enhancement is protein structure prediction, which plays a predominant role in interpreting the early stages of cancer. This article introduces a Protein Structure Prediction Technique (PSPT) based on Three-Dimensional Sequence (3DS). Figure 1 presents the proposed technique’s complete functional representation.

The ensemble model in this research combines diverse machine learning and deep learning algorithms to improve the accuracy of cancer prediction. It integrates various data types such as imaging, genomics, and clinical records. and uses a stacking approach to leverage the strengths of different models. The rationale is to mitigate biases, reduce overfitting, end enhance generalization to new data. The novelty lies in the specific design and application of the ensemble model for more effective cancer detection. The heterogeneous nature of cancer data encompass complex relationships and various modalities, makes the ensemble approach particularly suitable for capturing intricate patterns.

The integration of IoT in this research aims to enhance the precision of cancer prediction through three-dimensional sequence analysis of protein structures. IoT facilitates efficient data handling, retrieval, utilization and reorganizes cancer research with the contribution of clinical assessments. For enhancing data association PSPT based on 3DS utilizes IoT components such as computing and storage system.

The 3DS model demonstrates the concatenation of amino acids and their folds analyzed during the initial stages of cancer. By exploring these progressions and the inflammatory response score of the protein structure, it becomes feasible to speculate the consequences of cancer. Deep ensemble learning is engaged to determine the sequence and folding responses, which leads to improvements in inflammation prediction. To estimate the structure changes, the determined scores are correlated with clinical data for individual structures and their folds. Recurrent ensemble learning compares response scores and obtains matches or mismatches between sequences estimated in the protein structures.

A variant of machine learning methods called deep ensemble learning approaches are employed in this research for determining sequence and folding responses, enhances the inflammation prediction, and estimating structure changes from the protein structures that ultimately contributes to the improved cancer prediction and analysis. Deep ensemble learning generally involves combining predictions from multiple base models to improve overall performance.

The process of using the protein structure for the cancer detection process is explained by the following equation given below:

1 $$\left. \begin{array}{*{20}l} {\frac{d}{dt}G\left( {t;\alpha } \right) = f\left( {G,\alpha } \right)} \hfill \\ {G\left( 0 \right) = G_{0} } \hfill \\ {\begin{array}{*{20}l} {G = \left( {G_{1} , \ldots ,G_{d} } \right)} \hfill \\ {\alpha = \left( {\alpha_{1} , \ldots ,\alpha_{d} } \right)} \hfill \\ {\begin{array}{*{20}l} {\frac{d}{dt}G\left( t \right) = \frac{f\left( G \right)}{{f\left( \alpha \right)}}} \hfill \\ {\frac{d}{dt}G\left( \alpha \right) = f\left( G \right){*}f\left( \alpha \right)} \hfill \\ \end{array} } \hfill \\ \end{array} } \hfill \\ \end{array} \right\}$$

where $G$ is denoted as the protein structure, which is analyzed for further detection, $\alpha$ is represented as the variations in the protein structure, $t$ is denoted as the structural changes, and $f$ is represented as the dimension arrangements. The analyzing process is done by considering the characteristics mentioned earlier, which help in the cancer detection process.

The stacking method is used in this ensemble learning approach for combining predictions from different learning models that capture different patterns, improving generalization to different datasets, handling non-linear relationships in cancer data, and boosting overall predictive performance. The parameters include $Q$ repetition identification, relying on ${L}_{j}, \widehat{y}$, and $Q$ provided the $\Delta$ changes $\alpha$ based on the previous $Q$. The model enhances the robustness and accuracy of cancer prediction based on protein structures by utilizing the strengths of multiple models.

**Figure 2:**
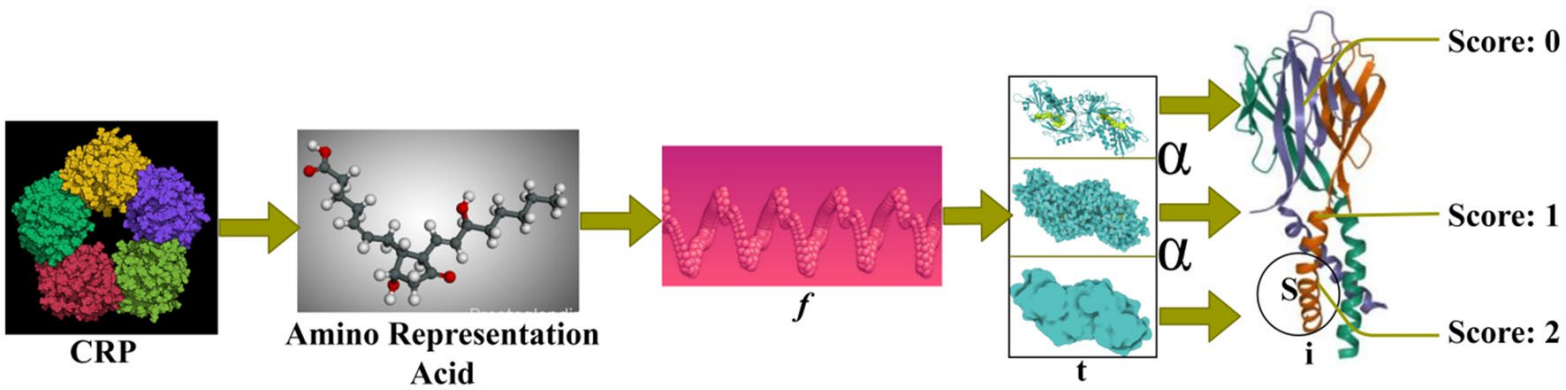
Mutation representations for score computation.

The amino acids extracted from the protein structure also help detect cancer in the human body. Amino acids, which are present in the protein structure, are their building blocks. Thus, it helps in the detection of cancer. The mutations and altered interactions in amino acids lead to abnormal protein structures associated with cancer. The patterns are determined by analyzing the consequences of acquired amino acids in the protein structure, which helps detect cancer in the human body. The process of using the amino acids in the protein structure for the detection of cancer is explained by the following equation given below:

2 $$\left. \begin{array}{*{20}l} {G^{\left( i \right)} = \left\{ {G\left( {t_{n}^{\left( i \right)} ;\alpha^{\left( i \right)} ,G_{0}^{\left( i \right)} } \right)} \right\}} \hfill \\ {where\;i = 1, \ldots ,N_{t} } \hfill \\ {\begin{array}{*{20}l} {n = 0, \ldots ,N^{\left( i \right)} } \hfill \\ {\left\{ {G\left( {t_{n}^{\left( i \right)} ;\alpha^{\left( i \right)} ,G_{0}^{\left( i \right)} } \right),G\left( {t_{n + 1}^{\left( i \right)} ;\alpha^{\left( i \right)} ,G_{0}^{\left( i \right)} } \right)} \right\}} \hfill \\ {\begin{array}{*{20}l} {where\;t = 0, \ldots ,N^{\left( i \right)} - 1} \hfill \\ {\left\{ {G\left( {0;\alpha^{\left( i \right)} ,G_{0}^{\left( i \right)} } \right),G\left( {\Delta_{t}^{\left( i \right)} ;\alpha^{\left( i \right)} ,G_{0}^{\left( i \right)} } \right)} \right\}} \hfill \\ {\begin{array}{*{20}l} {where\;t = 0, \ldots ,N^{\left( i \right)} - 1} \hfill \\ {\Delta_{n}^{\left( i \right)} = t_{n + 1}^{\left( i \right)} - t_{n}^{\left( i \right)} } \hfill \\ \end{array} } \hfill \\ \end{array} } \hfill \\ \end{array} } \hfill \\ \end{array}\right\}$$

where $N$ is denoted as the efficiency of the amino acids in the determination of cancer cells, $i$ is denoted as the mutations in the amino acids. The multiple folds of the protein structure are used to establish the inflammatory response score, which also helps extract the amino acid structure. The mutation representation of the considered proteins for score estimation is presented in Fig. 2.

**Figure 3:**
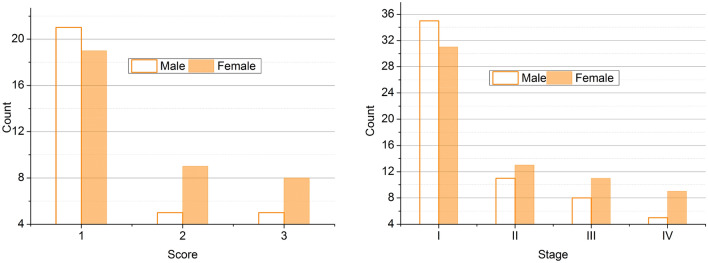
Score computed based on (male, female) and stages.

The CRP is represented by its amino acid structure from which $f$ is identified. For a varying $t$, the $\alpha$ between the $t$ is computed for classifying $S$ from $i$ provided the scores are computed. The maximum overlapping in $S \forall i$ results in high scores (abnormal), and the less $S$ results in low scores (normal). This is used for identifying scores from structural folds and clinical data correlations (Fig. 2). Based on the above, the representation of the score computation between males and females with four stages is analyzed in Fig. 3.

The computations presented above are validated based on the number of $t$ and the $\alpha$ between intervals. This computation is required to identify $S$ intensity in $i$. If this is high, the number of abnormalities is high, and therefore count increases. The stages (maximum abnormality) are defined based on the score. Therefore, the CRP response for tumor prediction is combined with the results in Table 1. Thus, the identifiable stages are augmented for $S\in i$ prediction (Fig. 3). The multiple folds in the protein structure are referred to in the three-dimensional arrangements to carry out the functions of detecting cancer. Multiple folds are observed in the initial stages of cancer, which helps determine the perception of the mechanisms. Understanding the various folds of the protein structures helps in considering the progression of cancer and also helps in establishing the inflammatory response score. Also, the knowledge of multiple folds aids in determining the efficacious therapies for further diagnosis. The process of understanding the multiple folds in the cancer detection process of protein structure is explained by the following equation given below:

3 $$\left. \begin{array}{*{20}l} {S = \left\{ {G_{j}^{\left( 1 \right)} ,G_{j}^{\left( 2 \right)} } \right\}} \hfill \\ {where\;j = 1, \ldots ,N} \hfill \\ {\begin{array}{*{20}l} {G_{j}^{\left( 1 \right)} = \left( {G\left( {0;\alpha^{\left( j \right)} ,G_{0}^{\left( j \right)} + \Delta j} \right)} \right)} \hfill \\ {G_{j}^{\left( 2 \right)} = \left( {G\left( {\Delta j;\alpha^{\left( j \right)} ,G_{0}^{\left( j \right)} } \right)} \right)} \hfill \\ {\begin{array}{*{20}l} {J\Delta = \mathop \sum \limits_{n = j} \Delta j} \hfill \\ {J:G \to G^{j} } \hfill \\ {\mathop \sum \limits_{n = 1} N\left( {G,J} \right) = \alpha_{n + 1} } \hfill \\ \end{array} } \hfill \\ \end{array} } \hfill \\ \end{array}\right\}$$

where $S$ is represented as the multiple folds in the protein structure,$J$ is denoted as the changes in the amino acid structures. By consolidating the efficiency of the amino acids $(N)$, their structural changes $(J)$, and protein structures, the multiple folds are analyzed to execute the response scores $\left(\sum_{n=1}N\left(G,J\right)={\alpha }_{n+1}\right)$. Using the deep ensemble learning technique, the inflammatory response score and the structural changes are extracted from the protein structures to enhance the inflammations. This deep ensemble learning technique also helps analyze the folds and their structure to produce the response scores further. It also determines the complex relationships of protein structure and protein folding data, aiding cancer detection.

4 $$\left. \begin{array}{*{20}l} {\begin{array}{*{20}l} {\hat{I} = \left[ {\begin{array}{*{20}c} {I_{d} } & 0 \\ \end{array} } \right]{ }} \hfill \\ {F = \left[ {\hat{I} + \hat{J}} \right]\left( F \right)} \hfill \\ {\begin{array}{*{20}l} {G\left( {G,\alpha ,dt} \right) = G + \hat{I}\left( {G,\alpha ,dt} \right)} \hfill \\ {S_{j}^{\left( 1 \right)} \to \left( {G,\alpha ,J} \right)} \hfill \\ {S_{j}^{\left( 2 \right)} \to \left( {G^{\left( j \right)} } \right)} \hfill \\ \end{array} } \hfill \\ \end{array} } \hfill \\ {\hat{N}\left( {S,\alpha ,dt} \right) \approx \Delta \left( {S,\alpha ,dt} \right)} \hfill \\ {\left( {S,\alpha ,dt} \right) \in I_{G} ,I_{\alpha } ,I_{\Delta } } \hfill \\ \end{array}\right\}$$

**Figure 4:**
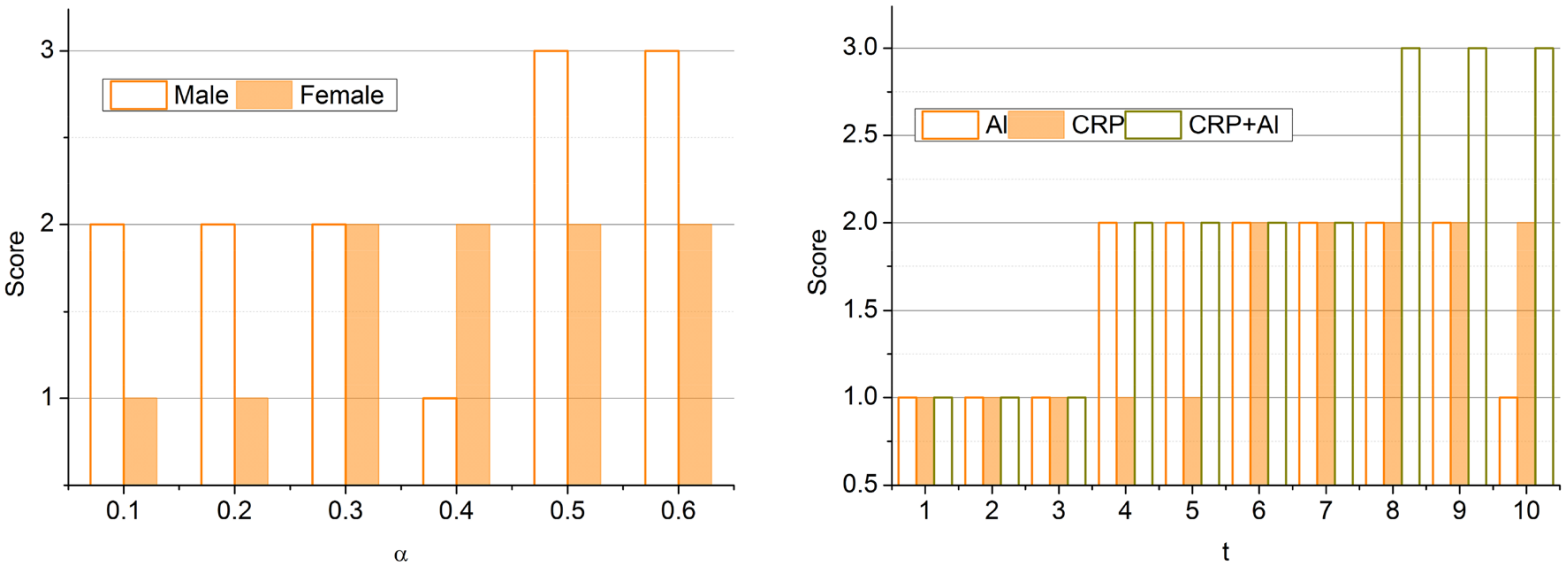
Response score classification analysis based on $\alpha$ and $t$.

The following equation above explains the process of using the deep ensemble learning technique to determine folds and their structure. The ensemble learning’s determination of the inflammatory response score and structural changes is now transpiring. The inflammatory response score is the criteria used in detecting cancer by using the protein structure. The inflammatory response score is estimated based on characteristics such as inflammation and cell activations using the multiple folds of the protein structures. This inflammatory score helps determine the quantity of inflammation in the body and aids in predicting cancer impacts. The process of extracting the inflammatory score from the amino acid structures is explained by the following equation given below:

5 $$\left. {\begin{array}{*{20}c} {\left\{ {\begin{array}{*{20}c} {R\left( {t_{0} ;\alpha } \right) = G_{0} } \\ {\hat{G}\left( {t_{n + 1} ;\alpha } \right) = \hat{G}\left( {t_{n} ;\alpha } \right)} \\ {t_{n + 1} = t_{n} + t_{j} } \\ \end{array} } \right.} \\ {J_{\alpha } \left( {G\left( {t;\alpha } \right)} \right) \approx J_{\alpha } \left[ {\hat{G}\left[ {t;\alpha } \right]} \right) = \smallint \hat{G}dG} \\ {I_{\alpha } \left( {G\left( {t;\alpha } \right)} \right) \approx I_{\alpha } \left[ {\hat{G}\left[ {t;\alpha } \right]} \right) = \smallint \hat{G}dJ} \\ \end{array} } \right\}$$

where $R$ is represented as the inflammatory response score detected from the multiple folds and amino acid structures. The response score classification for the $\alpha$ and $t$ is analyzed in Fig. 4.

**Figure 5:**
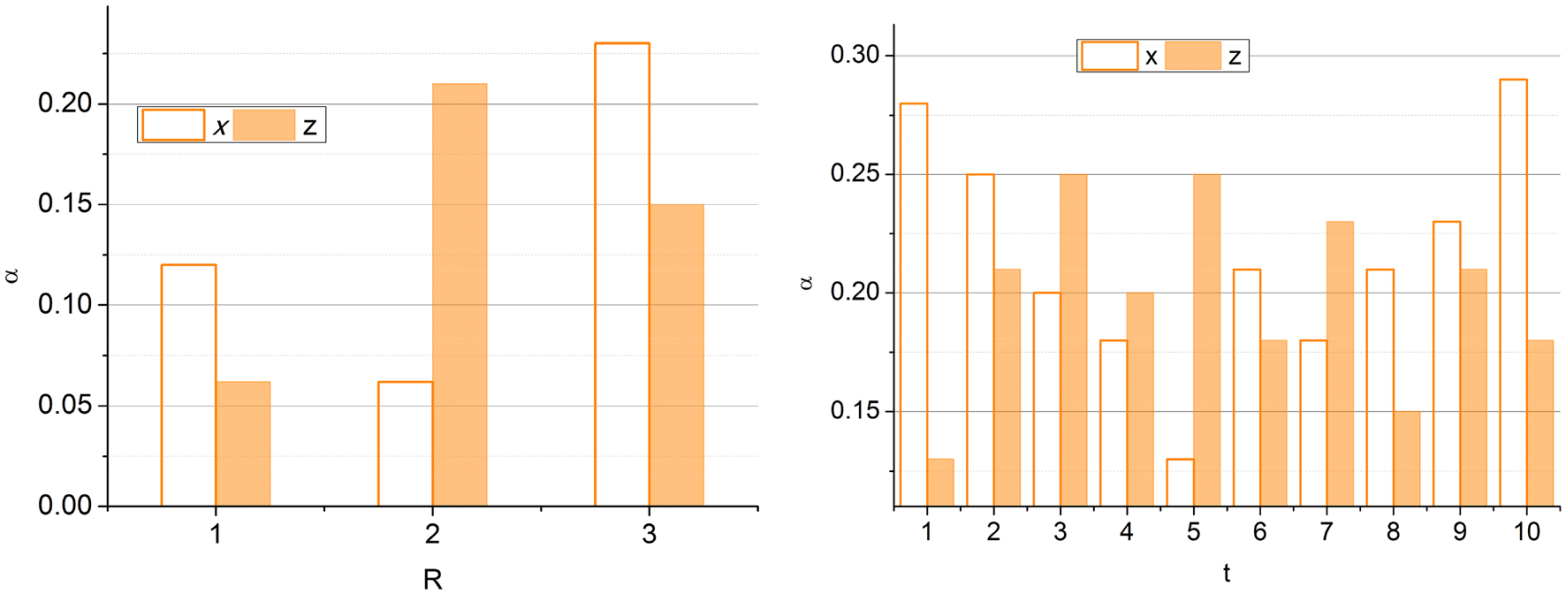
Variation estimation between *z* and *x*.

The score is analyzed for gender variation and protein variations. The maximum variation between successive $\alpha$ is $0.1$ to $0.6$, and the CRP and albumin proteins are considered. Based on the influencing factors, age, sex, smoking habits, and stage, the score is assessed. The change in variations results in high abnormalities, due to which the prognosis is observed for ${I}_{\alpha }\left(F\left(t;\alpha \right)\right)$ and ${G}_{o}$. Therefore, the cumulative variations are processed under undemanding $R$ (Refer to Fig. 4). The inflammatory response score’s level is now determined using deep ensemble learning. It verifies whether the estimated response score is high or low by analyzing the previous cancer prediction inflammatory score. This process helps assess the cancer level in the human body by considering the inflammatory response score. The process of analyzing the previous cancer prediction score for the verification process is explained by the following equation given below:

6 $$\left.\begin{array}{*{20}l} {\frac{dy}{{dt}} = - \alpha y} \hfill \\ {y\left( 0 \right) = y_{0} } \hfill \\ {\begin{array}{*{20}l} {y\left( {t;\alpha :y_{0} } \right) = y_{0}^{ - \alpha t} } \hfill \\ {G\left( {G\left( {t;\alpha } \right)} \right) = \mathop \smallint \limits_{0}^{1} d\alpha = \left( {\frac{{1 - j^{t} }}{t}} \right)^{2} } \hfill \\ {\begin{array}{*{20}l} {\mathop \sum \limits_{n = 1} \left[ {G_{i} ,t,\alpha } \right] = \frac{{1 - j^{2t} }}{t}} \hfill \\ { = \left( {\frac{{1 - j^{2t} }}{t}} \right)^{2} } \hfill \\ \end{array} } \hfill \\ \end{array} } \hfill \\ \end{array}\right\}$$

where *y* is represented as the previous prediction score. This previous cancer prediction score is used to verify the inflammatory score level in the detection of cancer procedures. By comparing the previous prediction score, the acquired score level is estimated. If the inflammatory score is high, it represents that the cancer cells are in more significant amounts in the body. If the inflammatory score is low, the cancer cells are low. This estimation process enhances diagnosis and helps boost the entire detection process. Thus, this process is explained by the following equation given below:

7 $$\left. \begin{array}{*{20}l} {\frac{d}{dt}\left( {\begin{array}{*{20}c} y \\ z \\ \end{array} } \right)\left( t \right) = \left( {W\left( t \right)\left( {\begin{array}{*{20}c} {y\left( t \right)} \\ {z\left( t \right)} \\ \end{array} + \alpha \left( t \right)} \right)} \right)} \hfill \\ {W\left( t \right) = \left( {\begin{array}{*{20}c} 0 & {S\left( t \right)} \\ { - S\left( t \right)} & 0 \\ \end{array} } \right)} \hfill \\ {\begin{array}{*{20}l} {\left( {W\left( t \right)\left( {\begin{array}{*{20}l} {y\left( t \right)} \hfill \\ {z\left( t \right)} \hfill \\ \end{array} + \alpha \left( t \right)} \right)} \right) = \left( {W\left( t \right)} \right){*}\alpha \left( t \right)} \hfill \\ {\frac{{d^{2} y}}{{dt^{2} }}\left( t \right) + \frac{dy}{{dt}}\left( t \right) = G\left( {y\left( t \right)} \right)} \hfill \\ {Z_{0} \left( {t,y} \right) = \left\{ {\begin{array}{*{20}c} {Z_{0,1} \left( {t,y} \right)} & {t \in \left[ {t_{0} ,t_{1} } \right]} \\ {Z_{0,n} \left( {t,y} \right)} & {t \in \left[ {t_{n - 1} ,t_{n} } \right]} \\ \end{array} } \right.} \hfill \\ \end{array} } \hfill \\ \end{array}\right\}$$

8 $$\left.\begin{array}{*{20}l} {F\left( 0 \right) = \left[ {\begin{array}{*{20}c} x \\ {G\left( x \right)} \\ \end{array} } \right]} \hfill \\ {y\left( t \right) = \left[ {\begin{array}{*{20}c} {S\left( t \right)} \\ {Z\left( t \right)} \\ \end{array} } \right]} \hfill \\ {\begin{array}{*{20}l} {\frac{dy}{{dt}}\left( t \right) = \left[ {\begin{array}{*{20}c} {S\left( t \right)} \\ {F\left( {t,S\left( t \right),Z\left( t \right)} \right)} \\ \end{array} } \right]} \hfill \\ {\frac{dx}{{dt}}\left( t \right) = y} \hfill \\ {\begin{array}{*{20}l} {\frac{dy}{{dt}}\left( t \right) = - x} \hfill \\ {y\left( 0 \right) \to y\left( T \right)} \hfill \\ {\begin{array}{*{20}c} {y:\left[ {0,T} \right] \to Z^{n} } \\ {\frac{df}{{dt}}\left( t \right) = F\left( {t,y\left( t \right)} \right)} \\ \end{array} } \hfill \\ \end{array} } \hfill \\ \end{array} } \hfill \\ \end{array} \right\}$$

where $z$ is denoted as the verification of the levels of the inflammatory score, $x$ is represented as the acquired level which helps in the detection of cancer. The structural changes are now extracted using the deep ensemble learning technique. The acquired inflammatory response score is associated with the clinical data protein structures and their folds independently to estimate the structural changes. Based on the acquired data and computed $R$, the variation estimation for prediction using the proposed technique is analyzed in Fig. 5.

The $\alpha$ observed from $x$ and $z$ are used for validating the stages of responses. The response score-based validations are performed using $x$ and $z$. The variations marked above represent the minimum and maximum $t$. This will be suppressed using an ensemble learning process for the changes observed in multiple sequences. Therefore, the structural folds are identified for $\alpha$ based on $\left(x,y\right)$ correlation, preventing the subsequent maximum deviation (Fig. 5). Comparing the predictions of the ensemble models in the detection of cancer operation helps in extracting and analyzing the detected structural changes in this procedure. This detection of structural changes helps in the diagnosis process with well-enhanced therapies and helps detect cancer cells on time without delay. The process of determining the structural changes by using the deep ensemble learning technique is explained by the following equation given below:

9 $$\left.\begin{array}{*{20}l} {P\left( n \right) = P\left( 0 \right) + \mathop \smallint \limits_{0}^{1} P\left( {y\left( S \right)dx\left( S \right)} \right){ }} \hfill \\ { = \mathop \smallint \limits_{0}^{1} \left[ {1{ }0 \ldots 0} \right]\left[ {\begin{array}{*{20}c} 1 \\ {*} \\ {\begin{array}{*{20}c} \vdots \\ {*} \\ \end{array} } \\ \end{array} } \right]ds} \hfill \\ {\begin{array}{*{20}l} { = \mathop \smallint \limits_{0}^{1} ds} \hfill \\ {P_{1} :\left[ {0,T} \right] \to N^{\left( j \right)} } \hfill \\ {P_{2} :\left[ {0,T} \right] \to N^{\left( i \right)} } \hfill \\ \end{array} } \hfill \\ \end{array} \right\}$$

**Figure 6:**
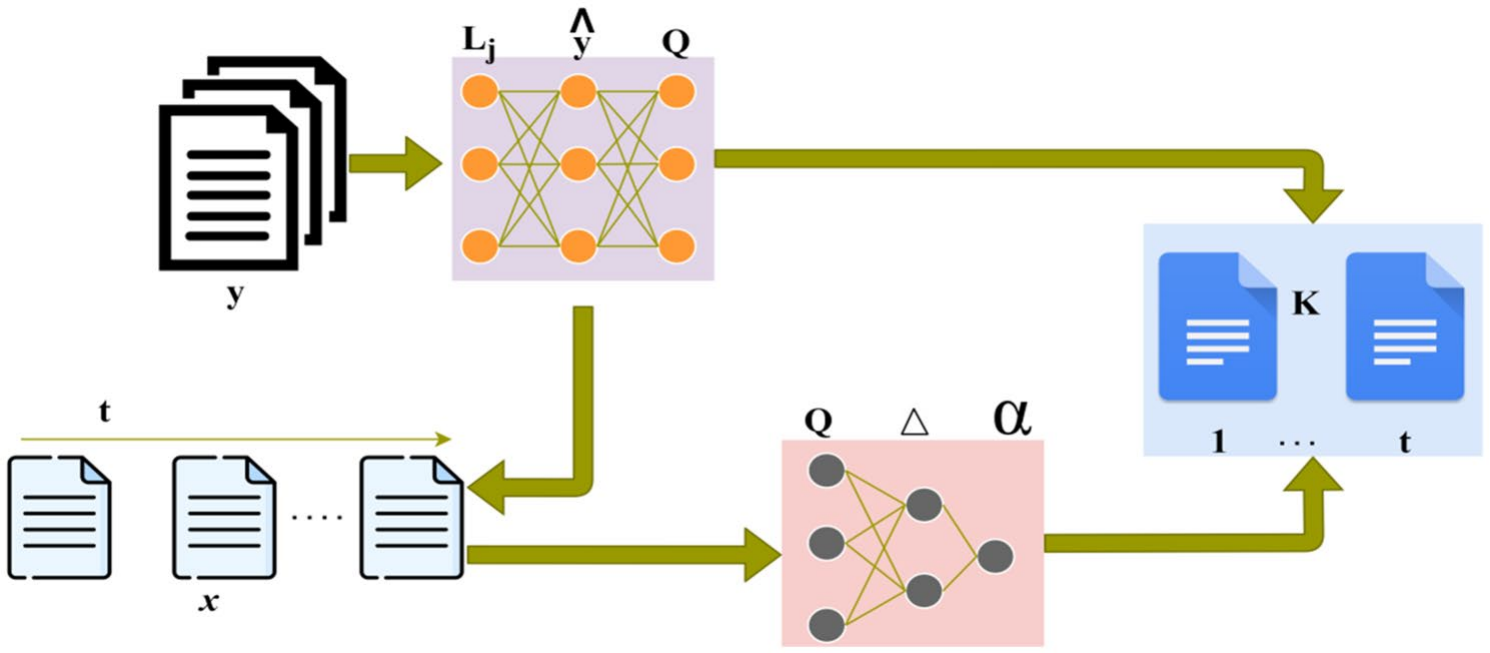
Learning process representations.

$P$ is denoted as the structural changes detected using the ensemble learning technique. The new processes commence with the estimated data if the structural change occurs. The process of starting the new process after the occurrence of structural change is explained by the following equation given below:

10 $$\left. \begin{array}{*{20}l} {K\left( {S_{j} } \right) = \left( {t_{j} ,y_{j} } \right)} \hfill \\ {K\left( {S_{j + 1} } \right) = \left( {t_{j + 1} ,y_{j + 1} } \right)} \hfill \\ {\frac{dK}{{dt}}\left( {S_{j} } \right) = \left( {\frac{{t_{j} - t_{j - 1} }}{{s_{j} - s_{j - 1} }},\frac{{y_{j} - y_{j - 1} }}{{s_{j} - s_{j - 1} }}} \right)} \hfill \\ {\frac{dK}{{dt}}\left( {S_{j + 1} } \right) = \left( {\frac{{t_{j + 1} - t_{j} }}{{s_{j + 1} - s_{j} }},\frac{{y_{j + 1} - y_{j} }}{{s_{j + 1} - s_{j} }}} \right)} \hfill \\ {where{ }s_{j} = t_{j} } \hfill \\ {X\left( S \right) = \left( {t_{j} ,y_{j} } \right) + \left( {t_{j + 1} - y_{j + 1} } \right)\left( {\frac{{S - S_{j} }}{{s_{j + 1} - s_{j} }}} \right)} \hfill \\ {for{ }S \in \left[ {S_{j} ,s_{j + 1} } \right]} \hfill \\ \end{array} \right\}$$

where ***K*** is denoted as the commencement of the new processes after the structural changes occur. As given in Eq. 10 above, the use of subscripts ***j, j − *1*, and j* + 1** suggests a parameterized sequence, possibly representing steps or intervals in a process. The subscript j in all places likely represents a specific point or value within a sequence represents a certain step or interval in a process. The subscript ***j − *1** represents the value immediately preceding the jth value. It is used to represent the previous step in the sequence. Similarly, the subscript ***j* + 1** represents the value immediately following the ***jth*** value thus making it easier to describe the behavior of a system or process over discrete steps or intervals. This paves the way for the study of time-dependent predictive features, which improve cancer prognosis prediction. Repeated ensemble learning occurs for matching and unmatching response scores to handle these structural changes. If the protein folds vary and do not match the response score, recurrent ensemble learning is done to enhance the precision of the prediction. The recurrent ensemble learning is done with the new data by analyzing the independent folding of the protein structures until it matches the previous cancer prediction inflammatory score’s response score. After these recurrent learning processes, the obtained inflammatory response score matches the previous response score at any instant. The process of repeated ensemble learning with new data is explained by the following equation given below:

11 $$\left. \begin{array}{*{20}l} {L_{j + 1} = L_{j} + F\left( {y_{j} } \right)\Delta t} \hfill \\ {L_{j} = L_{j + 1} - F\left( {y_{j} } \right)\Delta t} \hfill \\ {\begin{array}{*{20}l} {L_{j + 1} = L_{j} + \Delta t{ }} \hfill \\ {\Delta L_{j} = L\left( {t_{j} + 1} \right) - L\left( {t_{j} } \right)} \hfill \\ {\begin{array}{*{20}l} {\hat{y}_{j} + 1 = L_{j} - \hat{L}_{j} + y_{i} \Delta t} \hfill \\ {\Delta_{j + 1} = \Delta \left( {t_{j + 1} ,t_{j - 1} } \right)} \hfill \\ {y_{j + 1} = y_{j} + \frac{1}{2}\left( {\Delta j + \Delta_{j + 1} } \right)\Delta t} \hfill \\ \end{array} } \hfill \\ \end{array} } \hfill \\ \end{array}\right\}$$

12 $$\left. \begin{array}{*{20}l} {Q \le \left\{ {\left\{ {x,y} \right\}{|}x,y \in Z,{ }x \ne y} \right\}} \hfill \\ {x \in Z} \hfill \\ {\begin{array}{*{20}l} {\nabla \left( x \right) = \left\{ {y \in \frac{Z}{{\left\{ {x,y} \right\}}} \in Z} \right\}} \hfill \\ {Q\left( {S,t} \right)/Q\left( {S,j} \right)\sim N\left( {\frac{t - S}{{j - S}}} \right)} \hfill \\ {\begin{array}{*{20}l} {j = t{ }t \in Z} \hfill \\ {\Delta = \Delta_{j} + \Delta_{i} } \hfill \\ {\begin{array}{*{20}l} {t = \left( {S + j} \right)/2} \hfill \\ {L_{t} \sim \left( {S,t,i,j,Z} \right)} \hfill \\ \end{array} } \hfill \\ \end{array} } \hfill \\ \end{array} } \hfill \\ \end{array} \right\}$$

where ***L*** is denoted as the new data acquired from the protein structures, ***Q*** is represented as the repetition of the ensemble learning. The learning process is illustrated in Fig. 6.

The learning process retrieves $y$ and $x$ for identifying $\alpha$. This identification relies on ${L}_{j}, \widehat{y}$, and $Q$ provided the $\Delta$ changes $\alpha$ based on the previous $Q$. The t-based correlation for $x$ and $K$ identifies multiple ${L}_{j}$ and generates the chances of ${\Delta }_{i}+{\Delta }_{j}$. This is required for alleviating the $\alpha$ between successive t. Thus, in the correlation process, the three-dimensional representation of $t\in f$ is utilized for predicting $R$. Based on the $R$, the representations are computed for multiple J-preventing complexities (refer to Fig. 6). After these processes, the cancer type is predicted with the appropriate inflammatory response score. It also helps detect cancer stages by using deep ensemble learning. This repetition of the ensemble learning technique analyzes the independent handling of protein folds and inflammatory response scores for enhancing the prediction precision. Hence, the process of detecting the type and stages of cancer is explained by the following equation given below:

13 $$\left.\begin{array}{*{20}l} {\gamma \left( t \right) = F\left( {\gamma \left( t \right)} \right)} \hfill \\ {\frac{d\gamma }{{dt}}\left( t \right) = \frac{{F\left( {\gamma \left( t \right)} \right)}}{{Z\left( {\gamma \left( t \right)} \right)}}} \hfill \\ {\begin{array}{*{20}l} {\frac{d}{dt}\left[ {\begin{array}{*{20}c} x \\ y \\ \end{array} } \right]\left( t \right) = \left[ {\begin{array}{*{20}c} {\frac{\gamma \left( t \right)}{{1 + \gamma \left( t \right)}}} \\ {\frac{ - \gamma \left( t \right)}{{1 - \gamma \left( t \right)}}} \\ \end{array} } \right]} \hfill \\ {\frac{d}{dt}\left[ {\begin{array}{*{20}c} s \\ j \\ \end{array} } \right] = F\left( {S\left( t \right),j\left( t \right)} \right)} \hfill \\ {\begin{array}{*{20}c} {\left[ {\begin{array}{*{20}c} s \\ j \\ \end{array} } \right] \to \left[ {\begin{array}{*{20}c} {\frac{j}{S + j}} \\ {\frac{ - j}{{S - j}}} \\ \end{array} } \right]} \\ {where\;t \in \left[ {0,T} \right]} \\ \end{array} } \hfill \\ \end{array} } \hfill \\ \end{array} \right\}$$

where $\gamma$ is denoted as the prediction of the type of cancer and its stage. This proposed method helps detect the stage and type of cancer using deep ensemble learning. The precision of the prediction is efficacious in this method by reducing the complexity. The data correlation is enhanced in this cancer detection process and improves the structure change determination process. The acquired inflammatory response score level is detected by comparing the previous cancer prediction inflammatory response. The final prediction stage based on $\alpha$ detection is presented in Table 3. This representation is provided with changes between successive $t$.

**Table 3:** Prediction factor based on $\alpha$.

Stage	*t*	Value	*x* value	*R*	*α*	Precision
I	2	0.576	0.522	3	0.054	0.651
	4	0.596	0.589	3	0.007	0.819
	6	0.612	0.554	3	0.058	0.714
	8	0.654	0.571	3	0.083	0.519
	10	0.694	0.596	3	0.098	0.625
II	2	0.721	0.623	3	0.098	0.714
	4	0.689	0.589	3	0.099	0.614
	6	0.745	0.641	3	0.104	0.419
	8	0.692	0.694	2	0.002	0.841
	10	0.691	0.742	1	0.051	0.561
III	2	0.746	0.732	2	0.014	0.612
	4	0.774	0.693	3	0.081	0.698
	6	0.779	0.721	2	0.058	0.714
	8	0.754	0.697	3	0.057	0.652
	10	0.789	0.569	3	0.22	0.419
IV	2	0.714	0.598	3	0.116	0.514
	4	0.694	0.647	3	0.047	0.741
	6	0.621	0.698	2	0.077	0.869
	8	0.637	0.714	2	0.077	0.867
	10	0.749	0.697	3	0.052	0.745

The prediction precision for four stages with 2 t variations is in Table 3 above. The $y$ and $x$ are appropriate from the input data source and estimated. To be precise, this estimation is performed from the available $\Delta$ post-the ensemble process. This includes both correlation and $\alpha$ detection. In the $\alpha$ detection process, the sequential $t=2 to 10$ is considered. Therefore, the prognosis condition in Table 1 is validated for its abnormal/ normal observation for results. Thus, the deviations are estimated across different $S\in i$ for which $\alpha$ fluctuates. Thus, if it is less, precision is high, and vice versa. Thus, the identifiable learning outcomes are combined with $\left(\alpha , R\right)$ for this prediction.

The proposed methodology is compared with the recent state-of-the-art techniques to prove its superiority compared with techniques such as SWnet^28^, IDriveGenes^30^, and DNCL^24^ with the performance metrics like prediction precision, data correlation, structure change detection, correlation time, complexity, and accuracy.

## Discussion

The discussion is presented as a comparative study using the metrics prediction precision, data correlation, structure change detection, correlation time, and complexity. The observation sequences and response scores are varied accordingly in this analysis. The methods SWnet^28^, IDriveGenes^30^, and DNCL^24^ are considered in this comparative analysis.

### Prediction precision

**Figure 7:**
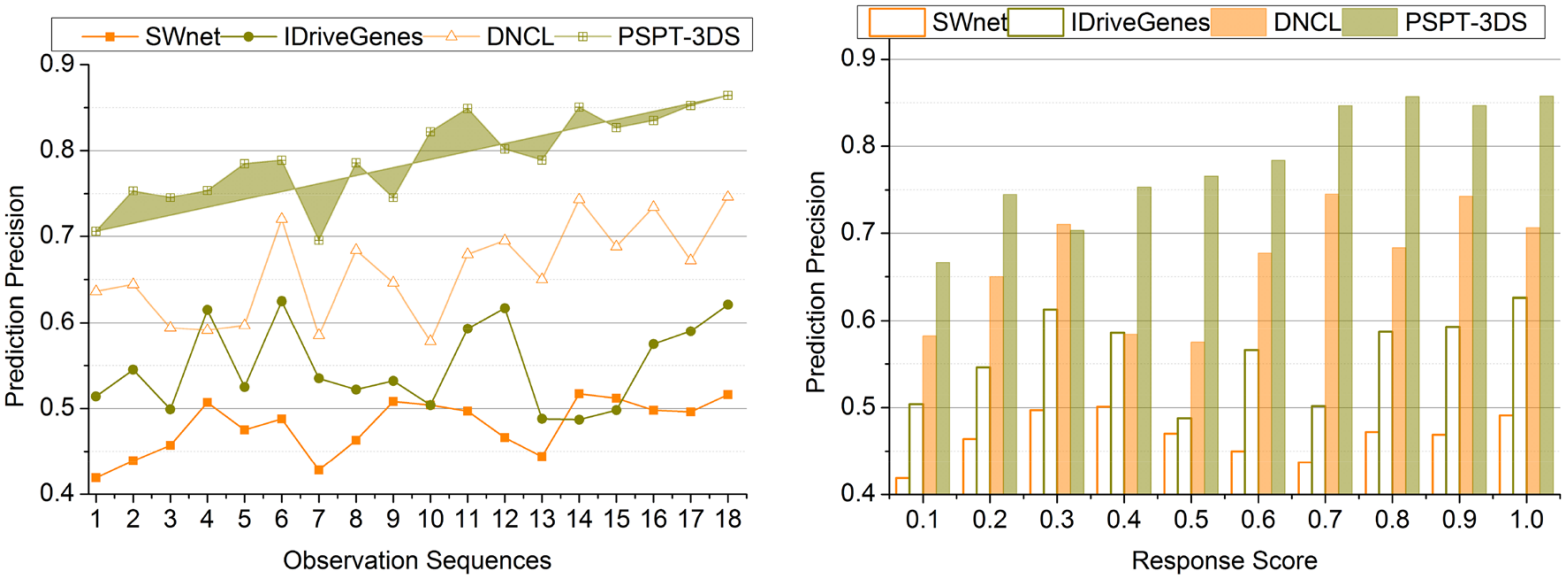
Prediction precision comparisons.

The precision of the prediction is efficacious in this process by using the deep ensemble learning technique to detect cancer. In cancer detection, the ensemble learning technique helps integrate various data, such as clinical data, inflammatory response scores, and protein structure data, to enhance prediction precision. This learning technique helps the acquired features determine the problematic patterns and relationships in the protein structures. The enhanced therapies are estimated for further diagnostic procedures with accurate cancer prediction using these models. The repeated ensemble learning process helps mitigate the high response score, making the precision more accurate. Thus, if the folds vary, the recurrent learning process is happening for predicting the cancer type and level of cancer cells. The multiple folds in the protein structures help determine the inflammatory response rate, which aids in verifying the cancer level in the human body. By consolidating these ways, this proposed system’s prediction precision is better (Fig. 7).

### Data correlation

**Figure 8:**
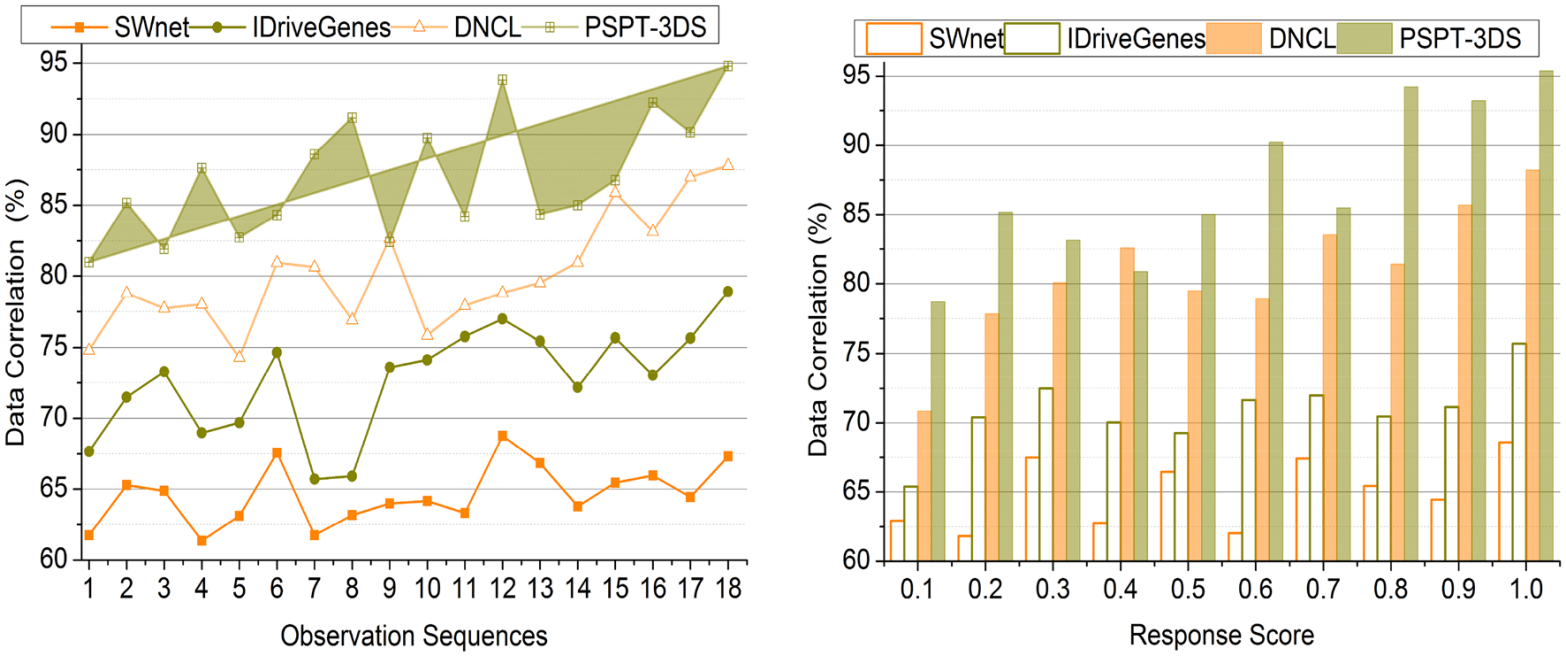
Data correlation comparisons.

The association of the data is better in this process with the help of the previous cancer prediction inflammatory response score. The correlation of the data is efficacious by comparing the previous clinical data with the acquired inflammatory response score. The data correlation is significant in detecting the process as it enables more understanding of the disease. Associating more data of protein structures and amino acids’ multiple folds helps develop the diagnosis of the cancer process. This enhanced data correlation helps determine the inducement of cancer and ids in detecting its type and stage. Better data correlation determines the progression of cancer and aids in the identification of risk factors. Improved data correlation also facilitates the association of the multiple structures, which leads to more precise and reliable predictions. The multiple folds in the amino acids determine the efficiency of the obtained inflammatory response score in detecting the cancer process (Fig. 8).

### Structure change detection

**Figure 9:**
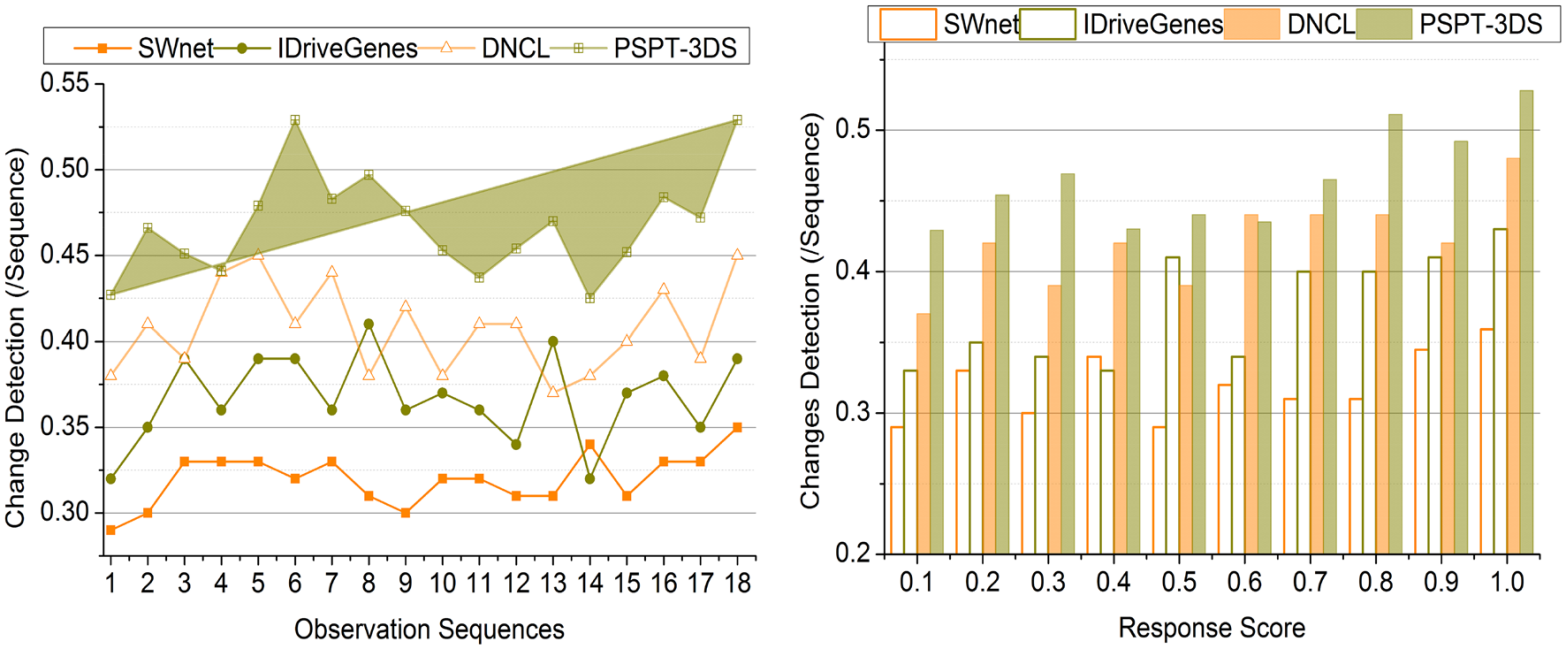
Structure change detection.

The structure change detection is efficacious in this process with the help of the deep ensemble learning technique. This ensemble learning technique in the detection of cancer aids in the better determination of the protein structural changes. This approach utilized multiple data which is associated with the development and progression of cancer. Hence, these characteristics are used to detect the structural changes during cancer detection and its stages. After estimating the diagnosis way and pattern to diagnose cancer, the level of the inflammatory response score is resolved. The ensemble learning technique helps identify the structural changes and inflammatory response scores. Depending on the inflammatory response score, the structural changes are evaluated to enhance the detection precision of the cancer. After predicting the changes in the protein structures, efficacious therapeutic strategies are estimated for the precise cancer diagnosis. By considering the ensemble learning process outcomes, the structure change detection is better in this process (Fig. 9).

### Correlation time

**Figure 10:**
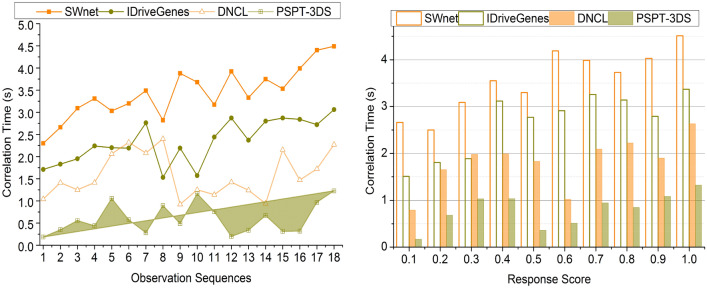
Correlation time comparisons.

The time taken for the data correlation is lesser in this process by using the amino acids and their structures in the protein structures. By analyzing the previous clinical data, the ensemble learning technique reduces the inflammatory response score and the time taken for the data correlation. The data correlation, such as consolidating the previous inflammatory response score, aids in detecting the level of the present acquired inflammatory response score. After estimating the high or low response score, the level of the cancer cells in the human body is identified. The protein structure is given as the input for the cancer detection process. Thus, the multiple folds of analyzing the amino acid structure and the changes in the protein structures are evaluated. If structural changes occur in the amino acids, the new process occurs according to the response score with the latest data from the protein structures. Thus, the correlation time is reduced by using these characteristics in the cancer detection process (Fig. 10).

### Complexity

**Figure 11:**
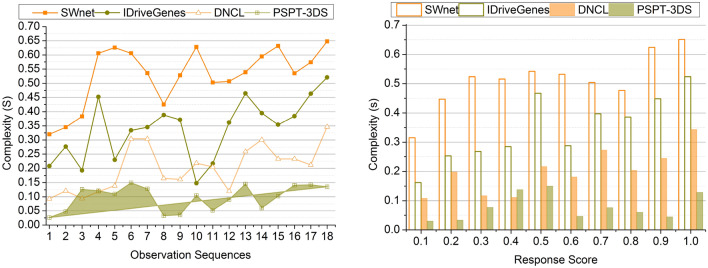
Complexity comparisons.

The deep ensemble learning technique reduces the complexities in this process. The protein structures play a vital role in the cancer detection procedure. This protein structure helps estimate the developed mechanisms to cure the cancer cells and helps develop the therapies that extract these protein structural abnormalities. The amino acids extracted from the protein structure also help detect cancer in the human body. The multiple folds in the protein structure are referred to in the three-dimensional arrangements to carry out the functions of detecting cancer. The deep ensemble learning technique also helps analyze the folds and their structure to produce the response scores further. It also determines the complex relationships of protein structure and protein folding data, aiding cancer detection. The inflammatory response score is the criteria used in detecting cancer by using the protein structure. The complexities are reduced by using the effective ensemble learning technique in the cancer detection process (Fig. 11). Finally, Tables 4 and 5 summarize the above discussion of the comparative analysis.

**Table 4:** Comparison results based on sequences.

Metrics	SWnet	IDriveGenes	DNCL	PSPT-3DS
Prediction Precision	0.516	0.621	0.746	0.8642
Data Correlation (%)	67.31	78.392	87.78	94.797
Change Detection (/Sequence)	0.35	0.39	0.45	0.529
Correlation Time (s)	4.49	3.06	2.27	1.226
Complexity (s)	0.648	0.521	0.346	0.1352
Accuracy (%)	69	77.5	86.9	96

*Findings:* The proposed technique improves prediction precision, data correlation, change detection, and accuracy by 11.83%, 8.48%, 13.23%, and 9.94% respectively. This technique reduces correlation time and complexity by 10.43% and 12.33%, respectively.

**Table 5:** Comparison results based on response score.

Metrics	SWnet	IDriveGenes	DNCL	PSPT-3DS
Prediction precision	0.491	0.626	0.706	0.8574
Data correlation (%)	68.59	75.71	88.21	95.376
Change detection (/sequence)	0.359	0.43	0.48	0.528
Correlation time (s)	4.51	3.37	2.63	1.321
Complexity (s)	0.651	0.524	0.343	0.1285
Accuracy (%)	71	78	87.5	97.3

*Findings:* The proposed technique improves prediction precision, data correlation, change detection, and accuracy by 12.49%, 8.94%, 10.5% and 10.62%, respectively. This technique reduces correlation time and complexity by 10.39% and 12.43%, respectively.

The proposed work involves classification methods, especially in the analysis of response scores for cancer prediction. It includes the classification of response scores based on gender variation, protein variations, and other influencing factors, leading to the assessment of abnormalities and the prognosis for various phases of cancer. For improving the overall performance of the model, accuracy is used as an additional metric.

### Accuracy

**Figure 12:**
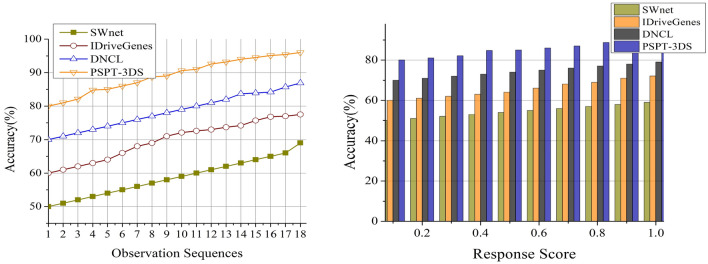
Accuracy comparisons.

Utilize distinct types of instances where the model correctly predicts the actual presence of cancer instances as true positive (TP) class. Likewise, in cancer prediction, where the proposed model correctly recognizes the non-cancerous are termed as true negative (TP). The third instance is false positive (FP) where the model incorrectly identifies the positive class where they are actually non-cancerous. False Negative (FN) are the instances where the suggested idea wrongly identifies instances as non-cancerous even though they are cancer (Fig. 12). Protein folding and response values can be assessed on an individual basis with the use of 3DS analysis. To improve the forecasting of sequence and folding responses, the system uses deep ensemble learning, which combines many models. Cancer detection is enhanced by an ensemble technique, which helps capture complicated correlations in structural protein information and folding data.

## Conclusion

This article introduced and discussed the three-dimensional sequence analysis for predicting the cancer sequences from protein structures. This technique allows for the individualistic analysis of protein folds and response scores, subscribing to more precise predictions. IoT computing and storage systems promote the data association procedure, which enables efficacious restitution and utilization of relevant information. By manipulating the power of IoT technologies, this proposed approach improves the precision of cancer prediction and analysis by providing valuable insights for clinical assessments. Therefore, integrating IoT and developing the PSPT based on 3DS have reorganized cancer research. This technique combines precise interventions to enhance accuracy and provides a knowledgeable approach to analyzing the protein structures and predicting their impact on cancer development. The proposed technique improves prediction precision, data correlation, and change detection by 11.83%, 8.48%, and 13.23%, respectively. This technique reduces correlation time and complexity by 10.43% and 12.33%, respectively.

The proposed study combines insights and approaches from various fields or domains, including medical science, computer science, and data analysis. It concentrates on protein structure prediction and cancer detection aided by IoT. This collaborative nature helps to ensure that the research can also expand for various aspects and challenges present in different domains. Making the research findings more reliable and improving its applicability across disciplines.

### Future directions

The future research direction aims to combine genomics, transcriptomics, and proteomics data to enhance the PSPT’s accuracy and provide a more understanding of cancer biology. Apply the PSPT to individual patient profiles by incorporating genetic variations and lifestyle information, aiming for more precise cancer predictions, and personalized treatment recommendations.

## Data Availability

Data will be provided through corresponding author.
